# Case report: Fourth branchial cleft cyst: a case of acute suppurative thyroiditis

**DOI:** 10.3389/fped.2023.1212767

**Published:** 2023-07-07

**Authors:** M. Eduarda Caseiro Alves, Ana Nunes, Júlia Galhardo

**Affiliations:** ^1^ Pediatric Department, Hospital Dona Estefânia, Centro Hospitalar e Universitário de Lisboa Central, Lisbon, Portugal; ^2^ Radiology Department, Hospital Dona Estefânia, Centro Hospitalar e Universitário de Lisboa Central, Lisbon, Portugal; ^3^ Unit of Pediatric Endocrinology, Centro Hospitalar e Universitário de Lisboa Central, Lisbon, Portugal

**Keywords:** pediatrics, congenital anomalies, fourth branchial cleft cyst, thyroiditis, thyroid

## Abstract

This case report presents a 4 year-old-female patient with a neck mass who was diagnosed with an infected fourth branchial cleft cyst with left thyroid lobe involvement through fistulation. The case emphasizes the importance of considering uncommon etiologies, such as congenital anomalies, as a differential diagnosis when evaluating pediatric neck masses. The patient was prescribed broad-spectrum antibiotics, which led to the regression of the mass and inflammatory signs. Close follow-up in endocrinology and otorhinolaryngology appointments was maintained, and after 7 months, hypoplasia of the left lobe was observed. Thyroid function was reevaluated, and after two years, no recurrences were noted. The case highlights the significance of a comprehensive examination and assessment of corresponding clinical features, which can significantly reduce the rate of misdiagnoses and achieve an individualized diagnosis.

## Introduction

Branchial cleft anomalies are the second most common cause of congenital neck masses in children, accounting for approximately 20% of cervical mass diagnoses (1, 2). Although frequent, 95% of these cases are attributed to second branchial cleft lesions, while third and fourth remnants are quite rare (1–3). These anomalies typically present as a cervical inflammatory process. In this report, we describe the case of a healthy child with an anterior neck mass causing airway compression and thyroiditis, resulting from an infected fourth branchial cleft cyst that communicated with the superior left lobe of the thyroid. This association is not uncommon, according to the available literature (4–7). However, due to the infrequent occurrence of these congenital malformations, they may not always be recognizable in clinical practice. This clinical case highlights the importance of prompt diagnosis and appropriate management of rare pathologies such as branchial cleft cysts.

## Case report

A previously healthy child presented to the emergency department with a progressively painful neck mass that had been noted two days prior to admission. The patient also complained of a non-paroxysmal dry cough, progressive dysphagia for solid foods, and dysphonia that had started nine days earlier. The mother also reported one episode of low-grade fever in the two weeks preceding admission. Further history was obtained from the mother, revealing that the parents were nonconsanguineous, the pregnancy was unremarkable, and the patient was born by vaginal delivery at full term. On examination, the patient had stable vital signs, and no fever was present. Physical examination showed a hard, lightly tender anterior neck mass measuring approximately 4 cm in width, with no associated lymph node enlargement.

### Diagnostic assessment

Laboratory testing at admission showed a white blood cell count of 12.5 × 109 cells/L (reference range 5 to 15 × 109 cells/L) with 42% segmented neutrophils, and C-reactive protein of 20,9 mg/L (reference range <5 mg/L). Thyroid-stimulating hormone (TSH) was 6,23 UI/ml (reference range is 0.35 to 5.50 μIU/ml) with free thyroxine (FT4) of 0.96 ng/dl (reference range is 0.90 to 1.60 ng/dl).

Soft tissue neck ultrasound (US) showed a left anterior cervical inflammatory mass, measuring 30 mm×15 mm, extending to the left lobe of the thyroid, suggesting an inflammatory process.

**Figure 1:**
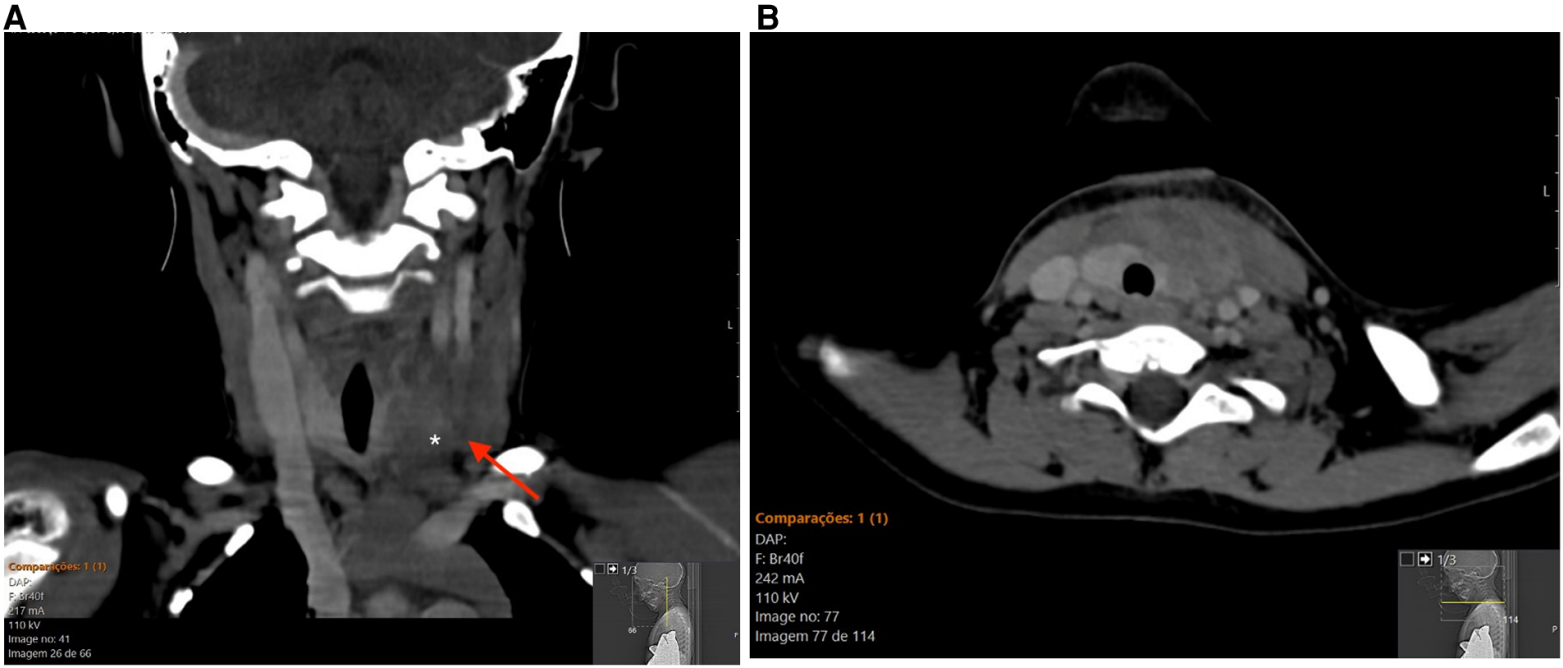
Contrast-enhanced CT of the neck. (**A**) coronal reformat displaying a ill-defined inflammatory mass (arrow) infiltrating the left lobe of the thyroid gland. (**B**) corresponding axial CT image.

Lateral neck radiography showed a slightly compressed airway. Computed tomography (CT) scan of the neck was performed and showed a ill-defined left anterior cervical inflammatory mass, measuring 30 mm × 15 mm, extending to the left lobe of the thyroid (Figure 1).

### Therapeutic intervention and follow up

The patient was prescribed broad spectrum antibiotic with amoxicillin and clavulanate 7:1 in the dose of 50 mg/Kg/day of amoxicilin every 8 h for 10 days and discharged home. After 7 days, physical exam showed regression of the mass and its associated inflammatory signs, as well as a resolution of the previously reported compressive symptoms.

After 16 days, blood tests showed a white blood cell count of 8,74 × 109 cells/L and C-reactive protein of 1,4 mg/L (reference range <5 mg/L). Thyroid studies revealed a TSH of 7,83 μUI/ml, FT4 of 0.92 ng/dl and a free triiodothyronine (FT3) of 4,48 pg/ml (reference range is 2.78 to 4.42 ng/dl). Anti-thyroid peroxidase (TPOAb), anti-thyroglobulin antibody (TgAb), TSH receptor antibodies (TrAb) were negative. US of the neck was repeated and indicated an infected 4th branchial cleft cyst with left thyroid lobe involvement through fistulation.

The patient received close follow-up in endocrinology and otorhinolaryngology appointments. After one month, thyroid function was reevaluated with TSH 3.63 μUI/ml, FT4 0,92 ng/dl, FT3 4.05 ng/ml (reference range is 2.78 to 4.42 ng/dl), presumably returning to baseline.

**Figure 2:**
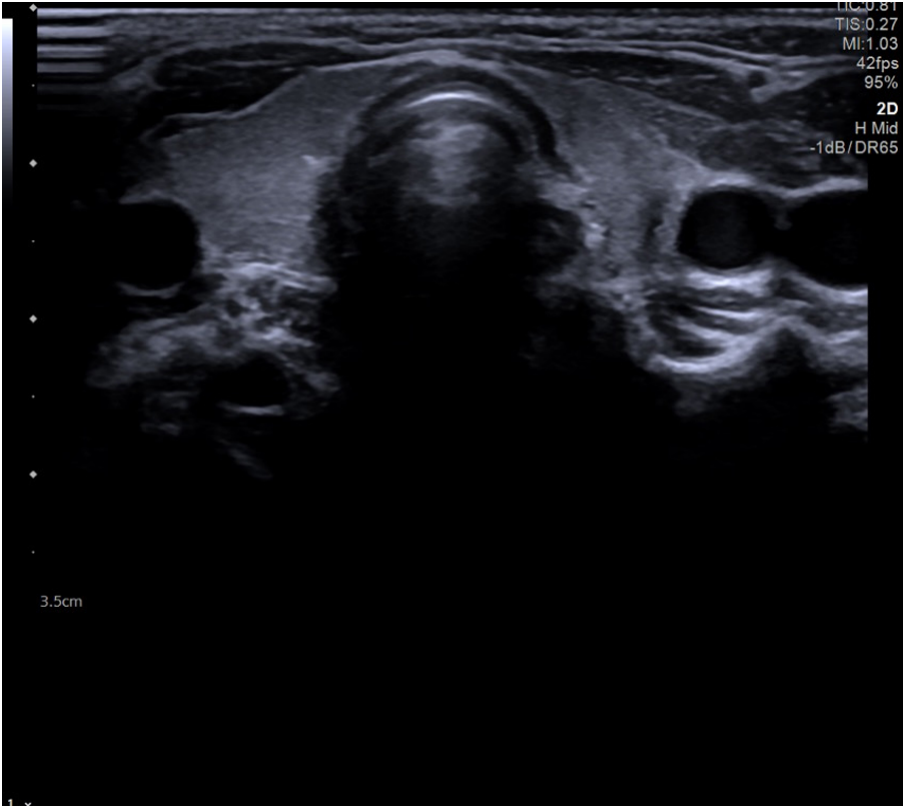
Thyroid gland assimetry with hypoplasia of the left lobe and structural heterogeneity.

After 7 months, a B-mode US of the neck was repeated and showed hypoplasia and heterogeneity of the thyroid left lobe (Figure 2).

After two years, no recurrences were noted.

## Discussion

Fourth branchial lesions are a rare cause of inflammatory neck masses and recurrent deep neck infections in children, particularly acute suppurative thyroiditis. These lesions occur due to abnormal embryological development of the branchial arches (8–10).

A fourth branchial cleft on the left side arises from the lateral neck and follows the path of the recurrent laryngeal nerve, looping around the aorta on the left and the subclavian artery on the right, and ending in the apex of the pyriform sinus near the cervical esophagus. This explains why fourth branchial cleft cysts can occur in various locations, including the thyroid gland and mediastinum (6, 10, 11).

This trajectory also explains our patient's complaints of hoarseness, which was due to recurrent laryngeal nerve inflammation, and selective dysphagia for solid foods, caused by narrowing of the cervical esophagus due to surrounding tissue inflammation.

Our patient presented imaging characteristics consistent with suppurative changes in the left thyroid lobe, along with a discrete elevation of the TSH. In most cases of acute suppurative thyroiditis, thyroid function remains uncompromised. However, some patients may develop transient thyrotoxicosis due to inflammation and destruction of the gland, while others may present with hypothyroidism, as shown by Callejo et al. (12).

Following the resolution of inflammation, thyroid function is expected to return to baseline. In our case, we did not have a baseline evaluation before the acute phase. However, by returning to reference range, one can assume the patient had no previous thyroid pathology. Autoantibodies were negative, ruling out immunological abnormalities.

The thyroid gland is well-protected against infections due to its capsular encasement, lymphatic drainage system, and high iodine and hydrogen peroxide content (13).

The left lobe, however, is more prone to infection due to its anatomical relationship with the fourth branchial cleft. Therefore, the presence of acute suppurative thyroiditis, especially on the left side, should prompt an investigation for an underlying branchial cleft sinus and fistula with the pyriform sinus (4, 13).

In a review of 526 cases by Nicoucar et al. (14) showed microorganisms susceptible to penicillin or related *β*-lactamase–resistant antibiotics to be the most frequent agents of infection. These findings support the use of broad-spectrum antibiotics targeting oropharyngeal flora microorganisms such as anaerobes, Staphylococcus aureus, Streptococcus pyogenes, Streptococcus epidermidis, and Streptococcus pneumoniae are the first-line treatment during the acute phase (15, 16).

In our case, our patient responded to the prescribed amoxicillin and clavulanate. Sanker et al. (17), also reported a case of acute suppurative thyroiditis caused by Klebsiella pneumoniae, sensitive to amoxicillin and clavulanate. In this case, a patient with diabetes type 2 presented with potential life-threatening situation, with multiple septic emboli and mediastinitis and urgent need for surgical drainage.

After the acute phase, fourth branchial malformations may require more definitive treatment to prevent recurrence. A thorough clinical examination should be conducted to evaluate the anatomical relation of the cyst. CT scans and MRI can be used to show the nature of the cyst and the anatomical relationship of the lesion (10, 11).

Regarding definite treatment, endoscopic cauterization may be attempted, especially in children under 8 years of age. This technique has a lower rate of complications but a higher recurrence potential if excision is incomplete (10). Alternatively, a more aggressive and definitive solution, with open neck surgery and hemithyroidectomy for complete excision of the tract, is an option for older children (6, 10).

In our patient's case, a watchful waiting approach was preferred due to the patient's age, maintaining close follow-up in otorhinolaryngology and endocrinology appointments, with no recurrence after two years. Although endoscopic or surgical excision is recommended to prevent further recurrences, some of these anomalies may be asymptomatic for years. In the case series of 526 pediatric patients shown by Nicoucar et al. (14), due to the high number of complications of surgery in patients younger than 8 years, the approach suggested is to reserve surgery for those above 8 years and maintain close follow up for recurrences after medical treatment. In another report, Bumber et al. (18) described a case a fistula which resolved spontaneously, suggesting a more conservative approach may be adequate in selected cases, even in older patients. MRI study was deemed unecessary for the time being after discussion with the Radiology team.

This case highlights the significance of including congenital anomalies, especially rare etiologies, as a potential differential diagnosis when assessing pediatric neck masses. It also illustrates how even atypical presentations may have an inflammatory nature. A thorough examination can help decrease the incidence of misdiagnosis, and by considering the relevant clinical features, we can attain a personalized diagnosis.

## Data Availability

The original contributions presented in the study are included in the article, further inquiries can be directed to the corresponding author.
